# Simplified screening and referral protocol for sinonasal mucormycosis in post COVID-19 patients

**DOI:** 10.1186/s42269-023-01032-x

**Published:** 2023-04-25

**Authors:** Jitendra Singh, Rubeena Arora, Vijay Rawat, Vikas Singh, Snigdha Goyal, Liza Joshi

**Affiliations:** 1 Department of ENT, Civil Hospital Panchkula, Block B, Room No. 11, Sector 6, Panchkula, Haryana 134109 India; 2 Department of Radiology, Civil Hospital Panchkula, Sector 6, Panchkula, Haryana India; 3 Department of Pathology, Civil Hospital Panchkula, Sector 6, Panchkula, Haryana India

**Keywords:** Mucormycosis, COVID-19, Rhinocerebral mucormycosis

## Abstract

**Background:**

To study incidence of sinonasal mucormycosis in active and post COVID-19 patients in a district-level hospital in India and develop a simplified screening and referral protocol for use at peripheral centres to aid rapid diagnosis/treatment.

**Methods:**

Study design: A prospective, interventional cohort study conducted from April 2021 to January 2022. Setting: Secondary level hospital in North India. Inclusion criteria: COVID-19 positive patients with diabetes mellitus as co-morbidity and with at least one of the following: received steroid therapy and/or on high flow oxygen therapy and/or had prolonged hospital stay (> 7 days). Exclusion criteria: Patients already immunocompromised/having malignancy/organ transplant recipients. Clinical workup: History, examination, imaging (CECT/MRI nose and paranasal sinuses if indicated), diagnostic nasal endoscopy + Nasal scrapings for KOH mount to detect fungal elements. STROBE guidelines were followed in the study.

**Results:**

Fourteen out of 250 patients tested positive for mucormycosis (incidence 5.6%). Thirteen were symptomatic, one patient was asymptomatic and detected on screening. No significant difference was found in mucormycosis versus non-mucormycosis group with respect to HbA1c status, vaccination status or steroid + oxygen treatment (*p* > 0.05 in all scenarios). Patients were treated with intravenous liposomal amphotericin B and surgical debridement when indicated. Two succumbed to disease (survival 85.7%). A clinical screening protocol was thus developed which can be used as an effective tool even at far-flung and remote healthcare facilities for diagnosis and timely referral of patients.

**Conclusions:**

Mucormycosis is a potentially lethal disease which needs rapid diagnosis and timely action to decrease morbidity and mortality.

## Key points

1. COVID causes immunosuppression.
2. COVID and diabetes mellitus together cause synergistic immune dysregulation.
3. Such patients are very vulnerable to develop opportunistic infections like Mucormycosis.
4. Our proposed screening protocol is easy-to-use even by minimally-trained healthcare workers.
5. Asymptomatic patients diagnosed with mucormycosis are relatively rare. Therefore, clinicians need to be vigilant of symptoms of ROCM.

## Background

The coronavirus pandemic, caused by the severe acute respiratory syndrome coronavirus 2 (SARS-CoV-2), has taken the world by storm and brought it to its knees. It has claimed 6.1 million lives worldwide, with 521,098 deaths reported from India, at the time of writing this report (WHO Coronavirus (COVID-19) Dashboard).

SARS-CoV-2 is responsible for a multitude of clinical symptoms, the most common clinical presentation being that of acute respiratory distress syndrome. It has also been associated with stroke, renal failure and other thromboembolic events, cardiomyopathy, coronary and systemic vasculitides, and new-onset diabetes mellitus due to its effect on the pancreas via ACE-2 receptors (Moorthy et al. 2021).

The mainstay of treatment in COVID-19 has been oxygen and steroids. Steroids are the only class of drug which has been found to be the most effective, with concrete evidence of significant decrease in mortality rate; other drugs have, at best, conflicting evidence of benefit with some even showing harmful effects (Sterne et al. 2020).

However due to the immunosuppressive nature of steroids, their increased use has led to an epidemic of opportunistic infections, one such opportunistic infection being rhinocerebral mucormycosis. Mucormycosis of the sinuses typically affects immunocompromised individuals such as those with uncontrolled diabetes mellitus, iatrogenic immunosuppression as with steroid use, AIDS, haematological malignancies and organ transplant recipients.

Clinically, rhinocerebral mucormycosis (ROCM) can present with signs and symptoms similar to complicated sinusitis, such as nasal blockade, crusting, proptosis, facial pain and oedema, ptosis, chemosis and even ophthalmoplegia. Headache, fever and neurological signs may be present if there is intracranial extension. Black discoloration/necrosis of tissues may be present but is not pathognomonic (Scheckenbach et al. 2010; Sharma et al. 2021).

In the current situation of COVID-19 pandemic, we have been seeing a disproportionately large number of cases of rhinocerebral mucormycosis, probably precipitated by the rampant increase in steroid use, both rational and irrational, in COVID-induced hypoxemia. Although mortality from ARDS has decreased thanks to steroid use, morbidity in the form of opportunistic infections especially rhinocerebral mucormycosis has risen.

Without early diagnosis and treatment, there is usually rapid progression of the disease, with mortality from intra-orbital and intracranial complications.

India is known as the diabetic capital of the world due to the high prevalence of this disease. This coupled with the COVID-19 pandemic and its accompanying unknown effects on the immune system, topped by injudicious steroid use, has resulted in an unprecedented increase in the number of mucormycosis cases, especially ROCM cases. Therefore, we conducted a study on the incidence of sinonasal mucormycosis in COVID-19 patients; both current and post COVID-19, at our district-level hospital.

## Aims and objectives

Early Screening and Diagnosis of Mucormycosis: To assess signs and symptoms of the disease at the earliest possible stage: key to successful outcome.

### Methods

#### Type of study

A prospective, interventional cohort study conducted at district-level hospital from April 2021 to January 2022.

### Ethical statement

The study was approved by Hospital Ethical Committee. All patients included in the study gave their written informed consent**.**

### Reporting guideline

The STROBE guidelines were followed in the preparation of this report.

### Inclusion criteria

All COVID-19 positive patients with diabetes mellitus as co-morbidity and with at least one of the following:

1. Received steroid therapy and/or
2. On high flow oxygen therapy and/or
3. Had prolonged hospital stay (> 7 days)

### Exclusion criteria

Patients:

1. Already immunocompromised
2. Having malignancy
3. Organ transplant recipients.

### Clinical workup

**Fig. 1:**
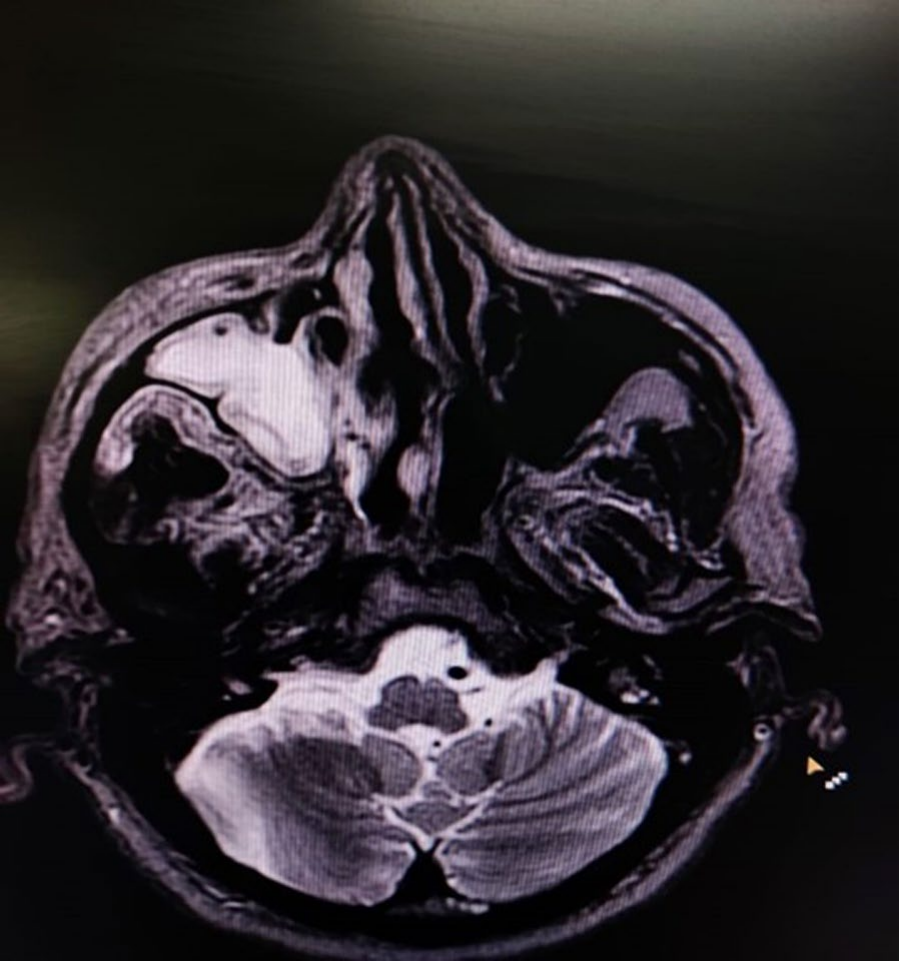
Contrast-enhanced MRI scan of nose and paranasal sinuses of a patient showing opacification of right maxillary sinus

All patients were subjected to a detailed workup including:

- Detailed medical and clinical history and examination
- Clinical symptoms and signs (warning signs and symptoms)
- Imaging studies in the form of CEMRI (Fig. 1), CECT nose and paranasal sinuses when symptoms/signs present
- Diagnostic nasal endoscopy (DNE) + DNE-assisted nasal swab/scrapings of suspected cases were taken for microscopy and staining
- KOH mount (90% sensitivity)
- Haematological investigations
- HbA1c

## Results

Patients showing clinical signs/symptoms of mucormycosis underwent imaging followed by diagnostic nasal endoscopy (DNE) and nasal swab/scrapings.

Two hundred and fifty patients fulfilling the above criteria were screened for mucormycosis using diagnostic nasal endoscopy (DNE) and deep nasal swab and/or nasal scrapings. Deep nasal swab was taken in 150 patients as they had no crusting; 100 got nasal scrapings from inferior or middle turbinate/septum/any other area of obvious crusting). The nasal scrapings thus collected were sent for immediate analysis for fungal elements using KOH mount.

Fourteen out of 250 patients came positive for mucormycosis on KOH mount (Table 1). Two came positive for Candida. Thus, the incidence of mucormycosis recorded in our study was 5.6%. Out of these 14 patients, 13 were symptomatic for mucormycosis. Only one patient who was asymptomatic had mucormycosis detected on screening with DNE and nasal swab. The number of symptomatic patients who came positive for mucormycosis on screening and imaging was 13. Out of the remaining 236 patients who were negative for mucormycosis, all were asymptomatic.

**Table 1:** Detailed data of patients

S. no.	AGE/SEX	h/o COVID positive to detection of mucor duration (DAYS)	Duration of hospital stay COVID-19 (days)	Diagnosis by	Clinical signs/symptoms	Treatment antifungals	Surgery	Other co-morbidity or immunocompromised status	Steroid + oxygen therapy received	Classification/category mucor
1	6/M	30	10	KOH mount + CEMRI	Nil (came + on screening F/b imaging)	Lp AmB i.v	N		Y	ROCM
2	59/M	45	12	KOH mount + CEMRI	Eye symptoms	Surgical debridement f/b Lp AmB i.v			Y	ROCM
3	60/M	30	10	KOH mount + CEMRI	Palate ulcer	Surgical debridement f/b Lp AmB i.v	Y	Nil	Y	ROCM
4	39/M	60	10	KOH mount + CEMRI	Nasal BLOCKAGE, crusting and pain + facial palsy	Surgical debridement f/b Lp AmB i.v	Y		Y	ROCM
5	52/M	60	11	KOH mount + CEMRI	Nasal BLOCKAGE, crusting and pain	Surgical debridement f/b Lp AmB i.v	Y		Y	ROCM
6	38/F	15	21	KOH mount + CEMRI	Palate ulcer	Surgical debridement f/b Lp AmB i.v	Y		Y	ROCM
7	35/M	15	15	KOH mount + CEMRI	Nasal BLOCKAGE, crusting and pain	Surgical debridement f/b Lp AmB i.v	Y	CAD + HTN	Y	ROCM
8	66/M	30	11	KOH mount + CEMRI	Eye symptoms	Surgical debridement f/b Lp AmB i.v	Y		N	ROCM
9	35/M	1.5	10	KOH mount + CEMRI	Eye symptoms	Surgical debridement f/b Lp AmB i.v	Y	CAD + HTN	Y	ROCM
10	61/M	60	8	KOH mount + CEMRI	Eye symptoms	Surgical debridement f/b Lp AmB i.v	Y		N	ROCM
11	48/M	45	Home isol	CEMRI	Eye symptoms	Lp AmB i.v	N		Y	ROCM
12	35/F	30	12	CEMRI	Nasal BLOCKAGE, crusting and pain	Surgical debridement f/b Lp AmB i.v	Y			ROCM
13	67/M	31	Home isol	KOH mount + CEMRI	Nasal BLOCKAGE, crusting and pain + eye	Surgical debridement f/b Lp AmB i.v	Y		Y	ROCM
14	49/M	29	16	KOH mount + CEMRI	Nasal BLOCKAGE, crusting and pain + eye	Surgical debridement f/b Lp AmB i.v	Y		Y	ROCM

*f/b* Followed by, *Lp AmB* Liposomal amphotericin B, *i.v.* Intravenous route

None of the 14 patients had symptoms of sinonasal disease prior to acquiring COVID.

Average age of our patients was 50.7 years (range: 35–67 years) with male sex predilection (12 out of 14 patients were male). All of these patients had pre-existing diabetes mellitus Type 2.

The average HbA1c of these 14 patients was 8.6% and of the remaining 236 patients was 8.4%; however, this difference was statistically insignificant (unpaired *T*-test, *p* = 0.93). However, these data do indicate that glycaemic control was poor in all our 250 patients.

It is notable that all 250 patients in our study had a history of prolonged hospital stay (> 7 days). Out of 250 patients, 186 patients received oxygen and steroids, steroid given was dexamethasone 6 mg orally for 10 days. No patient received steroid unless supplemental oxygen was required. Sixty-four did not require oxygen + steroids. (Table 2). In the 14 patients diagnosed with mucormycosis, 11 had received steroids + oxygen while 3 had not; while in 236 patients without mucormycosis, 175 had received steroids + oxygen and 61 had not (*p* = 0.7, not significant).

**Table 2:** Patient treatment details

	Received steroids + oxygen	Did not receive	Total
	175	61	236
	11	3	14
Total	186	64	250

	Vaccination status		
	One dose	Unvaccinated	
	97	139	236
	4	10	14
Total	101	149	250

A note on vaccination; 101 patients had received one dose of vaccine and 149 were unvaccinated (Table 2). Comparing the two groups (mucormycosis vs. non-mucormycosis patients) 4/14 and 97/236 had received a single dose (*p* = 0.35, not significant).

Our patients presented with the following symptoms; nasal symptoms (obstruction/bleeding/pain), facial pain, facial palsy, ophthalmic symptoms (eye pain/congestion), palatal necrosis/ulceration. Most patients had 1 or more of these symptoms: (detailed in Table 3).

**Table 3:** Patient symptom distribution

Symptoms	Number of patients	Ratio
Asymptomatic	1	1/14
Nasal symptoms	2 (Total)	2/14
Nasal obstruction	2	
Bleeding	1	
Pain	1	
Facial pain	9	**9/14**
Ophthalmic symptoms	5 (Total)	**5/14**
Eye pain	5	
Congestion	1	
Palatal necrosis/ulceration		2/14
Facial palsy		1/14

Bold values indicate the symptoms with highest frequency in mucormycosis patients

**Fig. 2:**
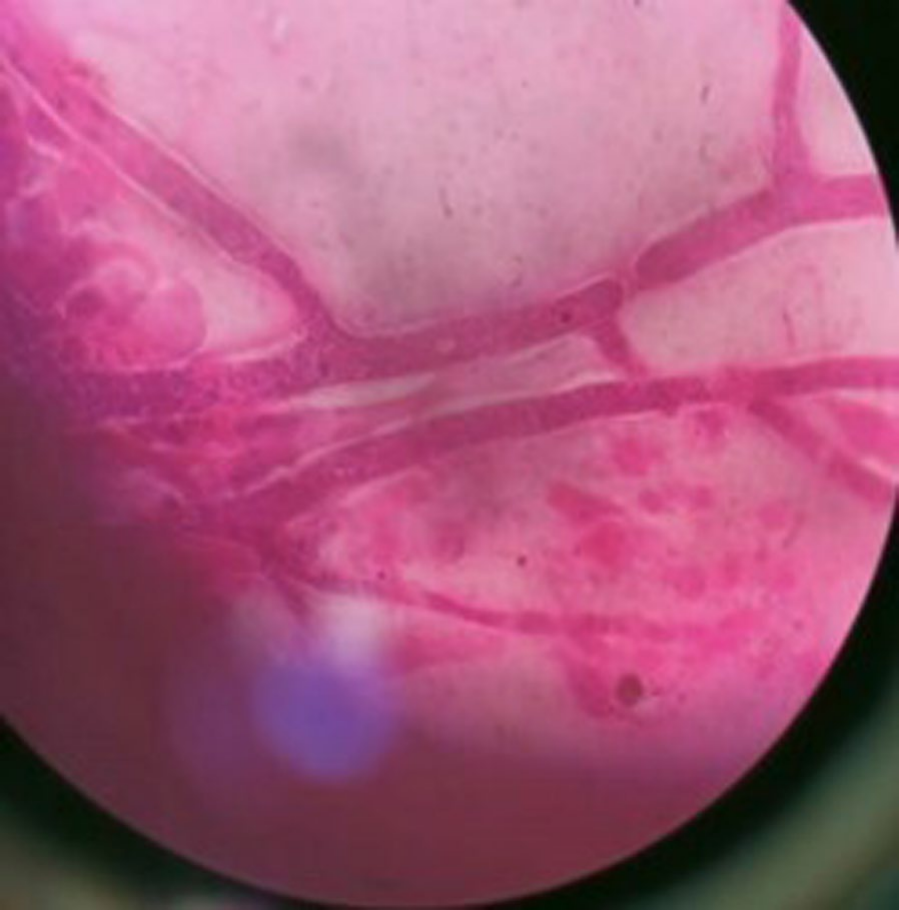
Histopathological examination of material from nasal scraping showing right-angle branching of aseptate hyphae-suggestive of mucormycosis (done after KOH mount)

All our patients underwent treatment in the form of intravenous liposomal amphotericin B for a total cumulative dose of 3 g, surgical debridement or both. Surgical debridement was done in indicated cases; 12 patients out of a total of 14 underwent intravenous liposomal amphotericin B + surgical debridement. Histopathological examination also turned out to be positive for mucormycosis in these 12 patients, thus reconfirming the initial diagnosis (multiple aseptate hyphae were seen branching off at right angles-Fig. 2).

Twelve out of 14 patients recovered with some sort of long-term sequelae. Two succumbed to complications of mucormycosis despite aggressive treatment. The details of these 14 patients are as per Table 1.

Surgical Interventions were performed at the nearest tertiary-care hospital. The procedures done were:

- Total Maxillectomy: 10
- Total Maxillectomy + Orbital Exenteration: 2
- Endoscopic Endonasal debridement: 2

Based on analysis of symptoms (Table 3), we conclude that facial and/or eye pain correlates most strongly with diagnosis of mucormycosis. So these symptoms must be specifically asked for and such patients should be referred immediately to a higher centre for further management.

## Discussion

COVID-19 pandemic has overstrained health systems all over the world. There has been unprecedented mortality and morbidity due to this disease. Moreover, most drugs have been found to be either ineffective or downright dangerous. The only drug, which has shown to reliably improve the course of COVID-19, is steroids. According to Indian guidelines, moderate cases should receive systemic methylprednisolone 0.5–1 mg/kg/day or dexamethasone 0.1–0.2 mg/kg for 3 days within 48 h of admission if inflammatory markers are raised or the need for mechanical ventilation is present. Severe cases should receive systemic methylprednisolone 1–2 mg/kg/day or dexamethasone 0.2–0.4 mg/kg for 5–7 days (Sen et al. 2021 Feb).

A multicentric study done by Patel et al. shows that sequential use of antifungal drugs, amphotericin B then posaconazole or isavuconazole, was independently associated with improved survival among mucormycosis patients. However, the optimal duration and dose of amphotericin B and posaconazole are not clear (Patel et al. 2021).

Mucormycosis is an opportunistic fungal infection. Factors which increase likelihood of this infection are diabetes mellitus, steroid use, any type of immunosuppression whether iatrogenic or otherwise, malignancies, etc. (Sen et al. 2021; Honavar 2021).

Mucormycosis can affect any organ with ROCM being the commonest manifestation (67% of total cases). There is a system for classification of ROCM (Honavar 2021) (Table 4). Worldwide pre-pandemic prevalence is 0.005–1.7 per million population, while prevalence in India is 0.14 per 1000 (Prakash and Chakrabarti 2019).

**Table 4:** Classification of COVID-19-associated rhino-orbital-cerebral mucormycosis (ROCM) as possible, probable and proven

Terminology	Definition
Possible ROCM	Typical symptoms and signs of ROCM Clinical setting of concurrent or recently treated COVID-19
Probable ROCM	Clinical features suggestive of ROCM Supportive diagnostic nasal endoscopy findings and/or Supportive radiological signs on contrast-enhanced magnetic resonance imaging or computed tomography scan
Proven ROCM	Clinico-radiological features suggestive of ROCM Microbiological confirmation on direct microscopy and/or culture and/ or histopathology with special stains and/or Molecular diagnostics

(Modified from Honavar SG. Code Mucor: Guidelines for the Diagnosis, Staging and Management of Rhino-Orbito-Cerebral Mucormycosis in the Setting of COVID-19. Indian Journal of Ophthalmology. 2021;69:1361–5.) (Sen et al. 2021 Feb)

Our incidence rate was 5.6% or 56 per 1000 population (14/250 patients). The respective estimated incidence for the following countries according to Prakash et al. are: 0.14 per 1000 (India), 0.0002–0.095 (Europe, Denmark and Portugal), 0.003 (USA), 0.0012 (Canada) and 0.0006 (Australia) (Prakash and Chakrabarti 2019). As is evident from these data, the incidence rate for India has jumped substantially in the COVID-19 pandemic.

All 250 patients had diabetes mellitus and had been COVID-19 positive at some point of time, and had one of the three risk factors as defined above. Only 14 came positive. So what was the common factor in our 14 patients that was not present in the remaining 236? The only differing factor was symptoms. Thus, a high index of suspicion based on the risk factors, along with a low threshold to perform imaging and nasal endoscopy + KOH smear is essential for early diagnosis. Many of our patients presented solely with facial pain (9/14). Our study suggests that in the absence of other concrete signs/symptoms and presence of risk factors, such patients should undergo imaging immediately followed by nasal endoscopy and KOH smears.

### Possible reason for such a high incidence rate

Ours is a large country with high population density. Moreover, India is known as the diabetic capital of the world with the second highest number of diabetics in the world (Honavar 2021). Patients here also have a tendency to self-medicate as many drugs are available over the counter, and many times access to a qualified doctor becomes difficult because of the huge population. Coupled with the severity of the second COVID-19 wave and rampant abuse of steroids, conditions were ripe for an epidemic of this otherwise rare infection. Some studies also suggest that COVID-19 causes hypoxia as well as deregulation of the immune system in such a way that growth of opportunistic organisms like mucormycosis is favoured (Sen et al. 2021; Honavar 2021).

Our protocol of intravenous liposomal amphotericin B was followed by 90 days’ of oral posaconazole 300 mg daily. Posaconazole was used both as a sparing agent and to offer additional antifungal cover in view of long-term side effects of liposomal amphotericin B.

Limitations of our study: Our sample size of 14 mucormycosis patients is relatively small to analyse positive effects, if any, of following up intravenous liposomal amphotericin B with posaconazole. Moreover, our focus was solely on mucormycosis and other fungal species were not analysed (2 candida spp, 1 aspergillus were detected as well but data were limited for analysis of these.)

Strengths of our study: The sample size which was screened was large consisting of 250 patients. Also our follow-up period of mucor patients was reasonably long with the average follow-up period being months (range 5–8 months.)

**Fig. 3:**
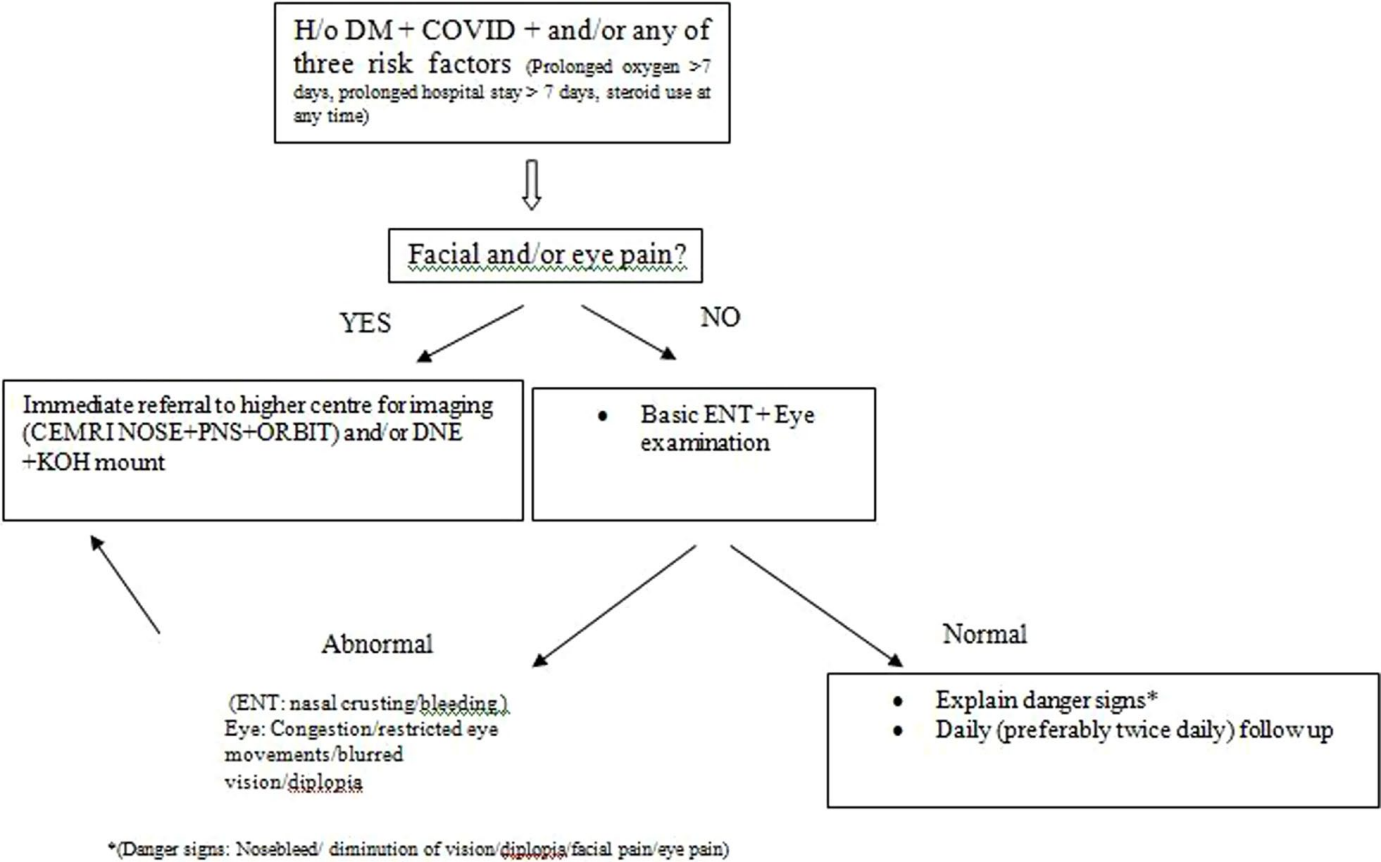
Proposed screening protocol on basis of clinical signs and symptoms which can be used in peripheral areas/areas with limited medical facilities. If a patient with diabetes and COVID and/or any 3 risk factors (as per flowchart) presents to the clinic, and they have facial/eye pain, immediately refer to higher centre for imaging and endoscopy. If no facial/eye pain, perform ENT examination—if it is abnormal, viz. nasal crusting/bleeding/eye symptoms—refer to higher centre as above; if ENT examination normal, explain danger signs and perform twice daily follow-up

Our findings therefore suggest that a screening protocol can be developed thus: (Fig. 3).

This screening protocol can be followed and implemented at peripheral medical centres and primary health centres. Healthcare workers at grassroots level can be trained accordingly so that they can immediately refer the patients for imaging and work up on basis of history and/or basic local site examination.

## Conclusions

Rhinocerebral mucormycosis (ROCM) is a potentially life-threatening condition which progresses rapidly. Its incidence has increased many folds in the COVID-19 pandemic. Adequately training our grassroots healthcare workers so that they can quickly diagnose such patients and refer them timely, can prevent morbidity and mortality.

## Data Availability

Data available on request.

## Abbreviations

COVID-19: Coronavirus disease
SARS-CoV-2: Severe acute respiratory syndrome coronavirus 2
AIDS: Acquired immune deficiency syndrome
ROCM: Rhinocerebral mucormycosis
ARDS: Acute respiratory distress syndrome
CEMRI: Contrast-enhanced magnetic resonance imaging
DNE: Diagnostic nasal endoscopy
KOH: Potassium hydroxide mount
CAD: Coronary artery disease
HTN: Hypertension

## References

[CR1] Sterne JAC, Murthy S, Diaz JV, Slutsky AS, Villar J (2020). Association between administration of systemic corticosteroids and mortality among critically Ill patients with COVID-19 a meta-analysis. JAMA.

[CR2] Honavar SG (2021). Code mucor: guidelines for the diagnosis, staging and management of rhino-orbito-cerebral mucormycosis in the setting of COVID-19. Indian J Ophthalmol.

[CR3] Moorthy A, Gaikwad R, Krishna S, Hegde R, Tripathi KK, Kale PG (2021). SARS-CoV-2, uncontrolled diabetes and corticosteroids-an unholy trinity in invasive fungal infections of the maxillofacial region? A retrospective, multi-centric analysis. J Maxillofac Oral Surg.

[CR4] Patel A, Agarwal R, Rudramurthy SM, Shevkani M, Xess I, Sharma R (2021). Multicenter epidemiologic study of coronavirus disease-associated mucormycosis. India Emerg Infect Dis.

[CR5] Prakash H, Chakrabarti A (2019). Global epidemiology of mucormycosis. J Fungi.

[CR6] Scheckenbach K, Cornely O, Hoffmann TK, Engers R, Bier H, Chaker A (2010). Emerging therapeutic options in fulminant invasive rhinocerebral mucormycosis. Auris Nasus Larynx.

[CR7] Sen M, Honavar SG, Bansal R, Sengupta S, Rao R, Kim U (2021). Epidemiology, clinical profile, management, and outcome of COVID-19-associated rhino-orbital-cerebral mucormycosis in 2826 patients in India: collaborative OPAI-IJO study on mucormycosis in COVID-19 (COSMIC), report 1. Indian J Ophthalmol.

[CR8] Sen M, Lahane S, Lahane TP, Parekh R, Honavar SG (2021). Mucor in a viral land: a tale of two pathogens. Indian J Ophthalmol.

[CR9] Sharma S, Grover M, Bhargava S, Samdani S, Kataria T (2021). Post coronavirus disease mucormycosis: a deadly addition to the pandemic spectrum. J Laryngol Otol.

[CR10] WHO Coronavirus (COVID-19) Dashboard https://covid19.who.int

